# Comparative Analysis of Energy and Exergy Performance of Hydrogen Production Methods

**DOI:** 10.3390/e22111286

**Published:** 2020-11-12

**Authors:** Angel Martínez-Rodríguez, Alberto Abánades

**Affiliations:** Energy Engineering Department, ETSII-UPM, Universidad Politécnica de Madrid, c/José Gutiérrez Abascal, 2, 28006 Madrid, Spain; abanades@etsii.upm.es

**Keywords:** hydrogen production, energy analysis, exergy analysis, hydrogen economy, hydrogen methods comparison

## Abstract

The study of the viability of hydrogen production as a sustainable energy source is a current challenge, to satisfy the great world energy demand. There are several techniques to produce hydrogen, either mature or under development. The election of the hydrogen production method will have a high impact on practical sustainability of the hydrogen economy. An important profile for the viability of a process is the calculation of energy and exergy efficiencies, as well as their overall integration into the circular economy. To carry out theoretical energy and exergy analyses we have estimated proposed hydrogen production using different software (DWSIM and MATLAB) and reference conditions. The analysis consolidates methane reforming or auto-thermal reforming as the viable technologies at the present state of the art, with reasonable energy and exergy efficiencies, but pending on the impact of environmental constraints as CO_2_ emission countermeasures. However, natural gas or electrolysis show very promising results, and should be advanced in their technological and maturity scaling. Electrolysis shows a very good exergy efficiency due to the fact that electricity itself is a high exergy source. Pyrolysis exergy loses are mostly in the form of solid carbon material, which has a very high integration potential into the hydrogen economy.

## 1. Introduction

The anthropogenic impact on global balance is reaching a level that endangers the development and well-being of humankind. The production and demand system that arose from the industrial era is unsustainable. Systemic changes are required to increase the sustainability of our society—in particular the energy sector—to avoid the collapse of its primary sources and raw materials, as well as drastic climatic modification, bringing uncertain consequences. The human impact on our global environment is focused on the combustion of fossil fuels producing large quantities of CO_2_ emissions, although there are many other structural processes on the metabolism of our society that have to be changed to pursue the implementation of a sustainable circular economy.

The reduction of greenhouse gases (GHG) emissions is a technological and social challenge of enormous dimensions, requiring the change from a society dominated by fossil fuels to a sustainable society through the use of fuels with low or no carbon emissions [1,2], thus complying with the indications of the Intergovernmental Panel on Climate Change (IPCC) [3].

Previous research shows hydrogen as an alternative vector for energy storage and its possible use as a fuel. The amount of energy produced by hydrogen combustion is higher than that produced by any other fuel on a mass basis with a low heating value, i.e., 2.4, 2.8 and 4 times higher than methane, gasoline and coal, respectively [4]. Hydrogen is the simplest and most abundant compound in the universe; however, it is not found in nature by itself, but is normally bound to oxygen or carbon molecules [5], so it must be obtained using different methodologies that will be analyzed.

The integration of hydrogen in the energy system is proposed either via distributed energy consumption and generation sites or centralized facilities [6]. Hydrogen can play a very important role in systemic changes as the implementation of Power-to-Gas schemes [7,8], that would help to manage electricity generation from renewable sources. The hydrogen provides solutions for improved, more flexible power grids. Storage options made possible by hydrogen technology offer new opportunities for self-consumption within a building, a city block or a small community, as well as new solutions for mobility. The challenge is to improve existing processes and replace fossil-fuel hydrogen with hydrogen from renewable resources whenever possible [9].

Advantages of hydrogen as a universal energy medium are that [10]:

1) The combustion of hydrogen results in the formation of steam and liquid water. In this respect, the use of hydrogen is practically safe from an environmental standpoint, compared to other combustion processes.
2) It is nontoxic.
3) It is easily assimilated into the biosphere; its combustion products are recycled by plants in the form of carbohydrates.
4) Hydrogen may be produced from the most abundant chemical on earth: water. Hydrogen can be obtained electrolytically, photoelectrochemically, thermochemically, by direct thermal decomposition, or biochemically from water.

Currently, 96% of the hydrogen produced worldwide comes from fossil fuels, mainly from reforming and gasification of natural gas, coal or naphtha for use in many industrial processes, such as ammonia production, and energy processes, such as in refineries or for direct consumption [11]. At present, a wide variety of hydrogen production processes are used, and can be divided into thermochemical methods, which consist of obtaining hydrogen from hydrocarbons, and methods related to the electrolysis of water, during which electricity is used to split the water into its constituent elements, hydrogen and oxygen. The inclusion of carbon capture and sequestration (CCS) in conventional fossil fuel-based hydrogen production processes offers another solution, but the cost increase associated with conventional CCS is high (40–100%) [12]. Other potential future methods of hydrogen production involving the conversion of sunlight into hydrogen by electrochemical cells or biological hydrogen production are being investigated at the basic science level [13].

Hydrogen can be produced from hydrocarbon fuels through basic technologies: natural gas pyrolysis (NGP), dry reforming of methane (DRM), steam reforming of methane (SRM), partial oxidation of methane (POM), autothermal reforming of methane (ATM) and other hydrocarbon reforming. These technologies produce a great deal of carbon monoxide (CO). Thus, in a subsequent step, one or more chemical reactors are used to largely convert CO into carbon dioxide (CO_2_) via the water-gas shift (WGS) and/or preferential oxidation (PrOx) [14]. These hydrogen production processes can be presented as a commercial mature technology which can be applied at low costs and achieve high efficiencies [15].

There are also other methods of hydrogen production without the use of hydrocarbons as fuel, such as electrolysis that uses water as raw input material or gasification that can use biomass. Table 1 shows an overview and brief description of some hydrogen production methods along with their primary energy and material sources.

Many compilations and comparisons have been conducted on different current hydrogen production technologies as shown in Table 1. Among the articles reviewing the state of the art and the various technologies of more recent hydrogen production more recent [17], you can see how research in this field is currently focused on finding technologies as productive as the most mature (reforming and gasification), using methods that are as environmentally friendly as possible. In addition, a wide range of raw materials for the production of hydrogen is being researched. These technologies still have challenges such as the total energy consumption and carbon emissions to the environment being too high [17].

We will focus our analysis on the most mature or promising for their implementation in the future, namely steam methane reforming (SMR) [18,19], partial oxidation of methane (POM) [20], coal gasification (CG) [21], auto-thermal reforming of methane (ATM) [22], water electrolysis (WE) [23], natural gas pyrolysis (NGP) [24] and dry reforming of methane (DRM) [25,26]. A more detailed theoretical description of each process can be found in the references cited. Table 2 shows a summary of this methods paying special attention to their CO_2_ production.

All these hydrogen production technologies have various advantages and disadvantages. These are the result of various parameters such as energy requirements, use or emission of harmful gases, costs, performance, or operating conditions. Table 3 shows the most significant advantages and disadvantages of each of the technologies on which this study focuses

Some authors have previously carried out studies on the comparison of different hydrogen production technologies based on different aspects: in general [28], according to economic feasibility and environmental efficiency [29], or from an energy or exergy profile but focusing on a specific technology [30]. The purpose of this article is to compare the different methods described previously for hydrogen production from an energy and exergy point of view to evaluate their constrains respect to their deployment conditions.

The difference between energy and exergy efficiencies is that energy or thermal efficiency uses only the heat source as input energy and in the case of exergy uses the fuel source as input energy. In short, exergy efficiency will always be relevant, and energy efficiency will only be relevant in cases where the energy source is in the form of heat. Therefore, this article is more relevant by focusing the comparison of these technologies beyond the energy profile and adding an exergy comparison, of which there is hardly any information in the literature.

For this work we have used contrasted parameters of working conditions, which provide high performance and efficiency in previous articles. This is due to the fact that this article tries to compare these technologies from the energy and exergy profiles always having in mind the most optimal working conditions for each technology and not trying to implement common conditions for the different technologies, since the purpose of the article is to determine which technology can reach higher efficiencies based on the compilation of previous recent studies on these.

This study will act as a tool to determine which technologies should be given higher priority in the development of their process, as they show the most promising values in energy and exergy efficiency for the continued study of hydrogen production as an energy source for the future.

## 2. Materials and Methods

To calculate the energy and exergy efficiencies of the different processes, several tools have been used following a common work pattern for all of them, always considering previous results of other investigations.

For the energy efficiency of processes, only the energies from the fuel and products (hydrogen or syngas) will be considered. This parameter is determined from the low heat value (LHV) and the input and output flows ($m_{i}$) Equation (1) [31]. In the case of exothermic processes (POM and WE) the heat of reaction $Q_{\mathrm{Heat}}$ is added as part of the product. Table 4 shows the low heat value (LHV) of pure substances.

(1) $$\eta_{Energy}=\frac{m_{prod}LHV_{prod}+\left(Q_{Heat}\right)}{m_{reac}LHV_{reac}}\cdot 100$$

Exergy efficiency is generally defined as the ratio of exergy recovered ($Ex_{out}$) to exergy supplied ($Ex_{in}$) [33].

(2) $$\eta_{Exergy}=\frac{Ex_{out}}{Ex_{in}}\cdot 100$$

Both parameters of Equation (2) are determined from Equations (3)–(6) [33].

(3) $$Ex_{in}=Ex_{fuel}+Ex_{Q}$$

(4) $$Ex_{out}=\sum^{\;}Ex_{prod}=\sum^{\;}n_{prod}Ex_{ch,prod}$$

(5) $$Ex_{fuel}=\sum^{\;}Ex_{reac}=\sum^{\;}n_{reac}Ex_{ch,reac}$$

(6) $$Ex_{Q}=Q_{Heat}\left(1-\frac{T_{0}}{T}\right)$$

where $Ex_{fuel}$ is fuel exergy, $Ex_{ch}$ are standard mole chemical exergy of pure substances, $n_{i}$ are molar flow, and $Ex_{Q}$ is exergy due to heat transfer. Table 5 shows the standard mole chemical exergy of pure substances.

Other important parameters of exergy are destroyed exergy and unused exergy, which are calculated with the following Equations [34].

(7) $$Ex_{Destruction}=Ex_{in}-Ex_{out}$$

(8) $$Ex_{Exhaust}=\sum^{\;}Ex_{subprod}=\sum^{\;}n_{subprod}Ex_{ch,subprod}$$

(9) $$Ex_{unused}=Ex_{Destruction}+Ex_{Exhaust}$$

The destroyed exergy measures the lost available energy that is unrecoverable. The unused exergy of the system is defined as the sum of the amount of exergy destroyed within the system and the amount of exergy wasted in the exhaust stream. The exergy of the exhaust stream is theoretically recoverable [35].

The processes have been simulated with the open-source process simulator DWSIM [36]. In addition, the mathematical software MATLAB was used to check the evolution of the different kinetics of each process (which were compiled from different previous articles): natural gas pyrolysis [37], dry reforming of methane [37], steam reforming of methane [38], partial oxidation of methane [39,40], electrolysis [41], coal gasification [42,43] and autothermal reforming of methane [44]. This information is detailed in “supplementary materials”. The same operating conditions, and kinetic equations have been introduced in both softwares to describe the evolution of the different components associated with each process. However, in the case of the DWSIM software, it is necessary to introduce a symbolic value very close to 0 as a reactor catalyst.

## 3. Results and Discussion

As shown above, the different processes discussed were simulated following the methodology described in the previous section. The results obtained are compiled according to various aspects for analysis and discussion

### 3.1. Fuel Conversion and Hydrogen (or Syngas) Production

Based on the kinetics of “supplementary materials” the chemical reactions that take place in the different processes were simulated, obtaining the conversions and products of these hydrogen production technologies. For the simulation of the different processes, previous articles have been taken as references, as indicated, since they show verified results, based on experimental cases, or are already used by several researchers, giving great validity to the kinetics used.

**Figure 1:**
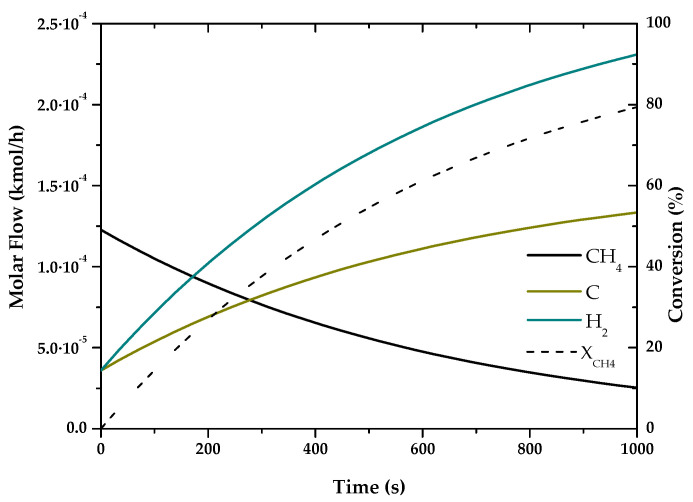
Evolution of molar flow profiles over time for Natural Gas Pyrolysis (T = 1173 K; P = 1 bar).

**Figure 2:**
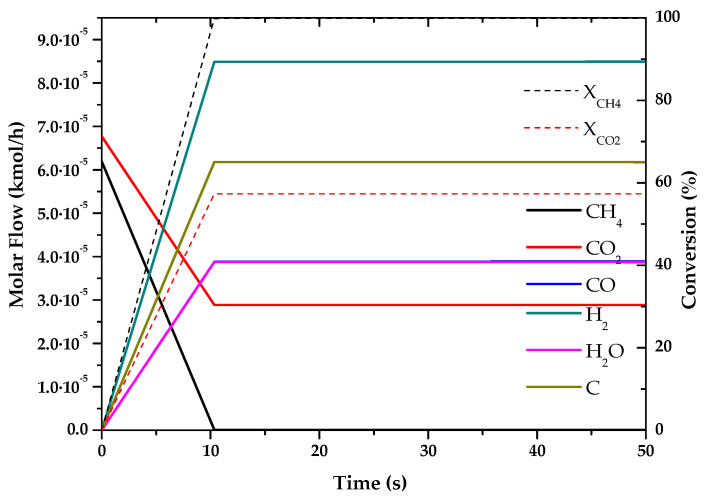
Evolution of molar flow profiles over time for Dry Reforming of Methane (T = 973 K; P = 1 bar).

**Figure 3:**
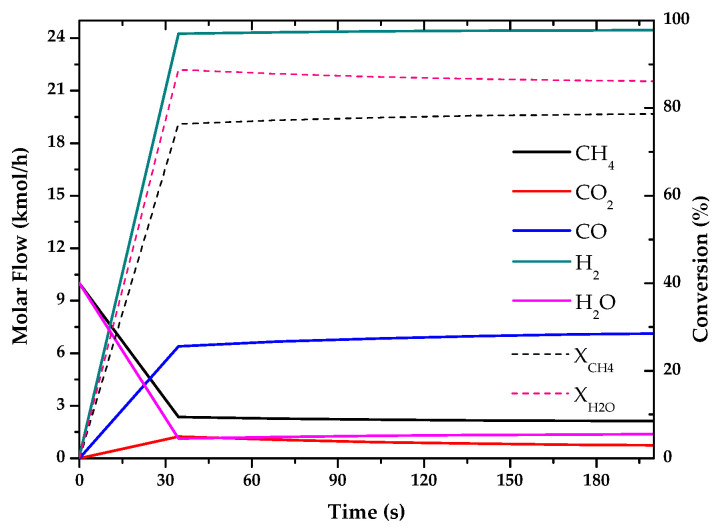
Evolution of molar flow profiles over time for Steam Reforming of Methane (T = 1000 K; P = 1 bar).

**Figure 4:**
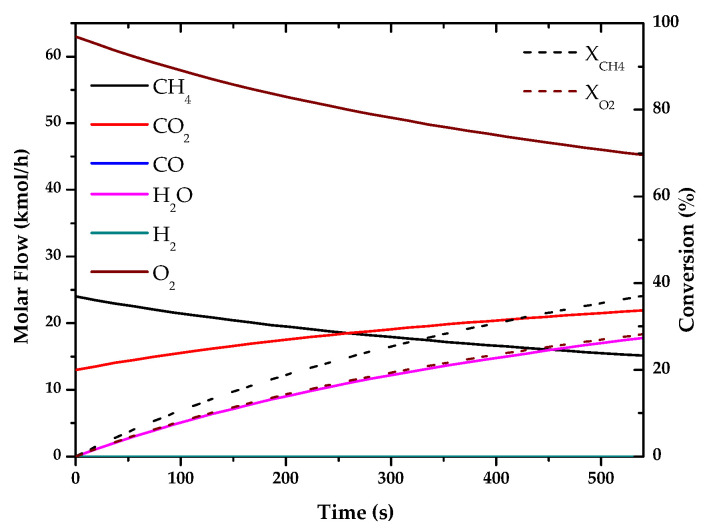
Evolution of molar flow profiles over time for Partial Oxidation of Methane (T = 1223 K; P = 1 bar).

**Figure 5:**
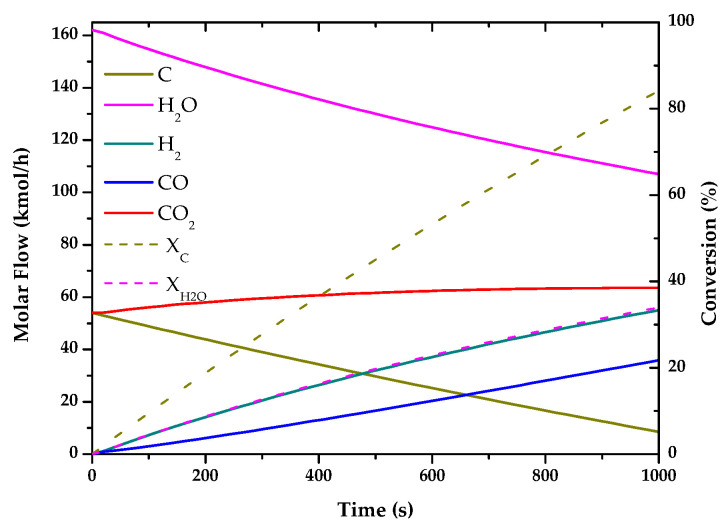
Evolution of molar flow profiles over time for Coal Gasification (T = 1123 K; P = 1 bar).

**Figure 6:**
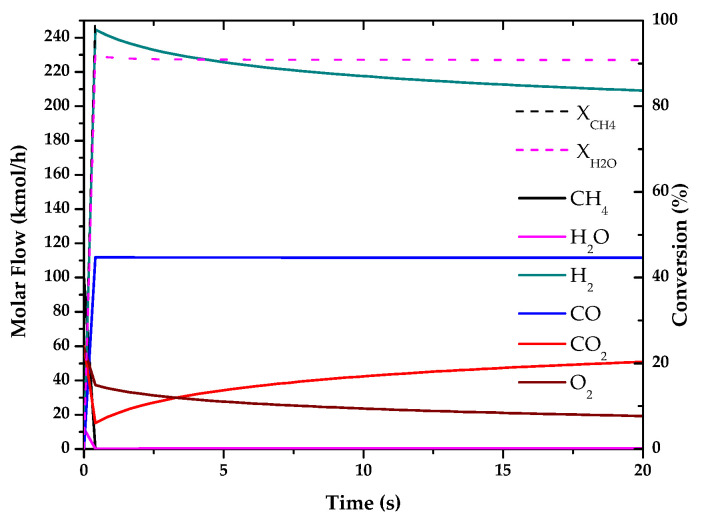
Evolution of molar flow profiles over time for Autothermal Reforming of Methane (T = 1510 K; P = 21 bar). The figures show the final molar flow results for each component involved in the reactions and fuel conversion, both as a function of the residence time to equilibrium, or to a fixed time known by the reference used for its simulation.

The results of these simulations are shown in the following graphs (Figure 1, Figure 2, Figure 3, Figure 4, Figure 5 and Figure 6). These simulations were carried out taking as operating conditions those described in the corresponding references for knowledge of kinetics.

In Figure 2, Figure 3 and Figure 6, it can be seen that the molar flow profiles deviate considerably at a certain reaction time. This phenomenon is attributed to the fact that the steady state is reached more quickly in these processes. This suggests that the dimensions of the reactor or the reaction time used for the simulation in our software do not match those used in the literature, but these parameters do not affect the subsequent calculations. This profile could be smoothed out by studying a greater number of points in the reaction time in the range where the steady state is reached.

With the simulations of the reactions that are carried out in each reactor, the kinetic constant for the consume of the main fuel for each one of the processes has been determined. In all these cases a simple order one kinetics has been assumed, adjusting the rate of fuel consumed to an exponential using the data analysis software OriginLab [45]. The results of the calculated kinetic parameters with operating conditions are shown in Table 6.

To produce hydrogen through thermochemical methods, a fuel with a hydrocarbon component in a gaseous state is used (except in coal gasification), and accompanied in some cases by other compounds such as CO_2_ (natural gas), H_2_O or O_2_. The products obtained after passing through the reactors are H_2_ and in some cases also CO, which is also partially converted to H_2_ through the water-gas exchange reaction (WGS). After these reactions, the products are separated from the rest of the compounds by means of a membrane system or any other separation operation. This separation system is another aspect to study in future investigations, however in this case similar values will be assumed for each process and therefore its influence is not of great importance for this study.

For water electrolysis, only electricity is used as a source of energy, as water cannot be considered as such. Its simulation was different from the rest of the thermochemical methods. This simulation was carried out only by using the DWSIM software with an EXCEL sheet, where an electrical power consumption of 18 kW was prefixed.

**Figure 7:**
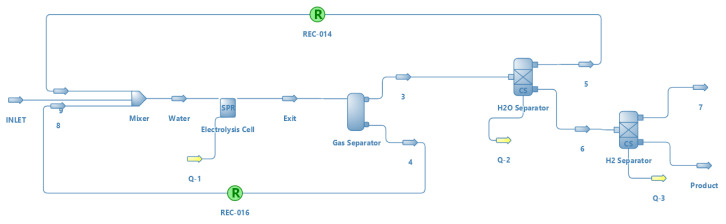
Simulation scheme for water electrolysis in DWSIM.

Figure 7 shows the simulation scheme for water electrolysis. The electrolysis cell is directly linked to the spreadsheet that simulates its function.

This figure also serves as a sample of the DWSIM software interface, and as an example for the rest of the processes simulated with this software in this study (supplementary materials).

### 3.2. Energy Analysis

Energy analysis is an important parameter for the viability of a process. Table 7 shows the energy efficiencies of each of the simulated processes. As these efficiencies are defined only by the reactants and products, they correspond essentially to the reactor efficiencies and not to the overall processes. The table compares the simulated results with DWSIM and MATLAB software in relation to the values of different references.

The thermal efficiency has been determined only for the transformation of raw materials into hydrogen and in the overall simulation process, where the energy required by the reactor is considered.

The energy output refers only to the energy of the products (hydrogen and carbon monoxide), and the energy loss includes the rest of the energy used in other process equipment such as pumps, electricity or separators. It should be mentioned that the output currents of the reactor at high temperatures have been used in all processes to heat the input current in a heat exchanger.

In general, the results obtained with the two software are practically identical (<5%), which gives validity to the kinetic models selected for each simulated hydrogen production method. This difference between the results of both software is due to the variation in the calculation method associated with the DWSIM software, but as the value is only 5% at maximum, the result and the software can be accepted as valid for the simulation of these processes, and possibly in new versions of this software the error will be reduced to a minimum.

In the case of water electrolysis, as indicated above, only its simulation was carried out with DWSIM. The comparison of these values with respect to the references is also positive, and the small variations in the values of the thermal efficiencies can be justified by the uncertainty of the complete kinetics used by the authors of these references. In general, the results of the energy efficiencies provide coherence to the simulations as was also observed with the molar flow profiles.

The consideration of the energy applied in the reactor can be estimated that in general in all the processes (except in the partial oxidation of methane, as it is an exothermic process), it reduces the efficiency between 15–30%.

Other parameters that have been calculated from these simulations are energy requirement, CO_2_ production depending on the production of H_2_, carbon-hydrogen selectivity and CO_2_ in relation to the carbon produced (Table 8).

In relation to the energy requirement of the processes, water electrolysis is the one that demands the most energy, about two times more than the second process (DRM). This is due to the greater amount of energy required to break the link of the water molecules than that of the hydrocarbons. In the same way, coal gasification has the lowest value because its fuel does not require breaking links for its reaction.

The rest of the parameters analyzed in Table 8, can be compared with the theoretical data in Table 2. The results show very similar data, except for partial oxidation, because stoichiometric do not consider the energy released by the hydrogen molecule. These results can be compared with those of other studies [46], obtaining data in accordance with ours. All the carbon produced in the processes is CO_2_, except in the pyrolysis of natural gas and in the dry reforming of methane where solid carbon is also produced.

The results of the energy balance show how the technologies of steam reforming of methane, water electrolysis and partial oxidation of methane, are the most efficient in terms of energy output. However, pyrolysis of natural gas does not produce CO_2,_ unlike all other thermochemical processes and the percentage of energy supplied to the reactor, which can come from renewable sources such as solar energy. Therefore, natural gas pyrolysis is a viable technology from the energy aspect, and really interesting as an alternative for hydrogen production.

### 3.3. Exergy Analysis

In addition to the energy balance, the exergy analysis of the different processes for the production of hydrogen was also carried out. For this exergy analysis the calculation of the efficiency was realized according to the process described below.

Exergy analysis can be used to improve process design by optimizing resources and operating parameters. Any excess heat in the process can be recovered as thermal or chemical energy, thus increasing the exergetic efficiency of the process [47].

The results of the exergy efficiencies are shown in Table 9, which contains the values obtained from the simulations carried out with the DWSIM software. These efficiencies, as well as the thermal efficiencies, are a function of the products in the reactor and not the overall process. They do not include compressor and pump exergy (low percentages of exergy in). The exergy efficiency is a parameter that is not so often studied as the energy efficiency, so it is complicated to compare it with other references. All percentages have been calculated based on the $Ex_{in}$.

The results show water electrolysis, autothermal reforming and steam reforming as are the methods with the highest exergy efficiency. In contrast, in methods such as coal gasification or dry reforming of methane, these values are over 1.6 times lower. This difference may be due to the presence of solid carbon in these processes, which contains high amounts of heat that without good optimization is wasted. Additionally, autothermal reforming is an energy self-sustained process, what implies a full utilization of the energy potential of the reactants. Water electrolysis has a high exergetic energy source as electricity with very low exergy destruction.

The natural gas pyrolysis showing lower exergy results than other more currently used processes such as steam reforming of methane. However, methane pyrolysis has the highest percentages of unused exergy, due to the by-product stream (solid carbon). As indicated above, the exergy associated with this parameter ($Ex_{Exhaust}$) is the only one that can theoretically be recovered, increasing the exergy efficiency of the process to double its value (~80%) [48]. In fact, solid carbon itself is a product that can be efficiently used in the context of the circular economy.

**Figure 8:**
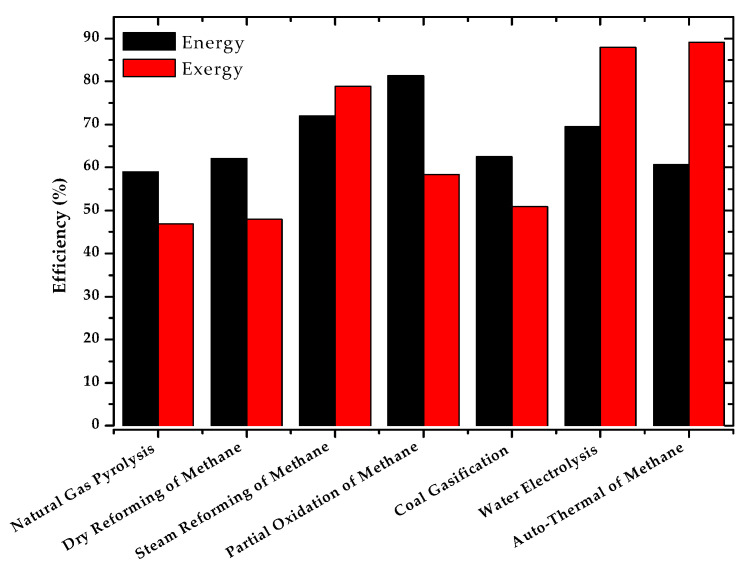
Comparison between energy and exergy efficiencies.

Figure 8 compares the values of energy and exergy efficiencies for the different simulated hydrogen production processes.

In view of the results, water electrolysis, steam reforming and auto-thermal reforming show the highest percentages. This demonstrates the fact that steam reforming and auto-thermal reforming are the most used technologies for hydrogen production at present. In the case of electrolysis, despite of being highly efficient in energy and exergy terms, its high energy demand (25 kWh/kgH_2_) makes this technology more expensive. However, it is a technology still under study with great expectations for the future.

The pyrolysis of natural gas is another technology with great expectations of study due to the commented possible increase of the exergy efficiency with the use of solid coal produced, and because it does not produce CO_2_. This means direct environmental benefits and indirect economic benefits, as no additional implementation of carbon capture and sequestration technology is required.

### 3.4. Carbon Capture and Sequestration/Storage (CCS)

A fundamental stage of the most used processes at industrial level today to produce hydrogen is the addition of CO_2_ capture and sequestration/storage (CCS) technology. Processes such as methane reforming, partial oxidation or autothermal reforming must be completed with a mechanism to separate and store the CO_2_ from its output streams, which greatly reduces its energy efficiency as well as increasing its costs.

For the implementation of this type of technology, various parameters must be studied, such as the specification of the quality of the captured carbon dioxide. In the literature, several critical issues in the transport part of carbon capture and storage chain have been identified and covered such as safety and toxicity limits, compression work, hydrate formation, corrosion and free water formation including cross-effects (e.g., hydrogen sulphide and water) [49].

Three technologies exist to capture CO_2_—pre-combustion, where CO_2_ is captured before fuel is burned; oxy-fuel, where CO_2_ is captured during fuel combustion; and post-combustion, where CO_2_ is captured after fuel has been burned (this technology can be retrofitted to existing power and industrial plants) [50].

This article did not study the different technologies or the characteristics of the CO_2_ streams, leaving it as a possible future research. However, assuming the implementation of post-combustion technology, which is the most employed in the industry, we know from previous studies that it carries a penalty of between 20–30% of energy efficiency. To make a more global comparison, a 25% energy efficiency penalty has been applied to all processes studied where additional implementation of carbon capture and sequestration is required. Table 10 shows the results with and without CCS technology.

The implementation of CCS means that technologies such as carbon gasification and dry reforming of methane are penalized in their overall energy efficiency. However, natural gas pyrolysis and electrolysis would increase their competiveness respect to mature technologies such as steam reforming, autothermal reforming and partial oxidation. Reducing the energy penalty associated with CO_2_ capture is one of the key issues of CCS technologies. The efficiency of carbon capture must be improved to reduce the energy penalty because the capture stage is the most energy-consuming stage in the entire process of CCS [31].

## 4. Conclusions

We have made an analysis of the most promising hydrogen production methods. Natural gas steam reforming is the preferred technology in many ambitious hydrogen deployment projects. Among the available fossil fuel based methods at an industrial scale, steam reforming shows the lower CO_2_ production per hydrogen product at a reasonable hydrogen cost. Nowadays, that is the preferred option in combination with carbon capture and sequestration (CCS) for high scale projects. In opposition, water electrolysis is a CO_2_-free method for hydrogen production, providing that clean electricity is used. Nevertheless, energy requirements for hydrolysis is 4 times higher than steam reforming, which implies a much more energy intensive processes with a strong dependency of the regulation and costs of the renewable electricity market and much more stress on the capacity of renewable energy facilities.

From the exergy analysis, dry reforming of methane and coal gasification, even if they are mature processes, show a lower efficiency. In particular coal gasification has higher CO_2_ production, which implies that in spite of the economic aspects, it is handicapped in its application to the energy transition towards a more sustainable system. The implementation of additional CCS techniques in the current state of the art would make coal gasification uncompetitive in the long term. Dry reforming is a process that shows a similar net CO_2_ emission as steam reforming, with the ability of reducing CO_2_ to CO at some point in the process, which could increase its interest in the context of the circular economy.

Our analysis is referred to as the basic reactor and associated process. The impact on the energy efficiency of the overall integration of hydrogen production technologies with a significant production of CO_2_ should be complemented with the consideration of CCS for their implementation into a sustainable future. In that sense, electrolysis and natural gas pyrolysis will not be strongly affected. There are other issues that should be analysed for the complete picture, as induced emissions by natural gas leakage, or life cycle of electrolyser components. Nevertheless, these technologies have to face the challenge of being able to achieve very high capacity of hydrogen production. In particular, natural gas pyrolysis must advance on its development. The production of carbon as main by-product and its full integration in the circular economy would increase the interest of methane pyrolysis as it will require much less energy than electrolysis with the additional outcome of a useful material for further processing (C).

## Figures and Tables

**Table 1:** Overview of hydrogen production methods by primary energy and material source [16].

Method	Source		Brief Description
	Primary Energy	Material	
Electrolysis	Electrical	Water	Direct current is used to split water into O_2_ and H_2_ (electrochemical reaction)
Plasma arc decomposition	“	Fossil fuels	Cleaned natural gas is passed through plasma arc to generate H_2_ and carbon soot
Thermolysis	Thermal	Water	Thermal decomposition of water (steam) at temperatures over 2500 K
Thermochemical (Water splitting)	“	Water	Cyclical chemical reactions (net reaction: water splitting into H_2_)
Thermochemical (Biomass conversion)	“	Biomass	Thermocatalytic conversion
Thermochemical (Gasification)	“	“	Conversion of biomass into syngas
Thermochemical (Reforming)	“	“	Conversion of liquid biomass (biofuels) into H_2_
PV electrolysis	Photonic	Water	PV panels are used to generate electricity
Photocatalysis	“	“	Water is split into H_2_ by using the electron-hole pair generated by the photocatalyst
Photoelectrochemical method	“	“	A hybrid cell simultaneously produces current and voltage upon absorption of light
Dark fermentation	Biochemical	Biomass	Biological systems are used to generate H_2_ in the absence of light
High temperature electrolysis	Electrical + Thermal	Water	Electrical and thermal energy are used together to drive water splitting at high temperatures
Coal gasification	“	“	Conversion of coal into syngas
Fossil fuel reforming	“	“	Fossil fuels are converted to H_2_ and CO_2_
Biophotolysis	Photonic + Biochemical	Biomass + Water	Biological systems (microbes, bacteria, etc.) are used to generate H_2_
Photofermentation	“	“	Fermentation process activated by exposure to light
Photoelectrolysis	Electrical + Photonic	Water	Photoelectrodes and external electricity are used to drive water electrolysis

**Table 2:** Hydrogen production methods and stoichiometric parameters ^1^.

	Global Reaction	$\frac{mol\;CO_{2}}{mol\;H_{2}}$	$\frac{mol\;C}{mol\;H_{2}}$	$\frac{mol\;CO_{2}}{mol\;C}$
Natural Gas Pyrolysis	$CH_{4}\;↔C+2H_{2}$	-	0.5	0
Dry Reforming of Methane	$2CH_{4}+CO_{2}+2H_{2}O↔2CO_{2}+6H_{2}+C$	0.33	0.50	0.5
Steam Reforming of Methane	$CH_{4}+2H_{2}O↔4H_{2}+CO_{2}$	0.25	0.25	1
Partial Oxidation of Methane	$3CH_{4}+2O_{2}+2H_{2}O↔3CO_{2}+8H_{2}$	0.38	0.38	1
Coal Gasification	$2C+4H_{2}O+CO_{2}↔4H_{2}+3CO_{2}$	0.75	0.75	1
Water Electrolysis	$H_{2}O↔H_{2}+0.5O_{2}$	-	-	-
Autothermal Reforming of Methane	$3CH_{4}+2O_{2}+2H_{2}O↔8H_{2}+3CO_{2}$	0.38	0.38	1

^1^ CO Syngas is assumed to produce CO_2_ by water shift reaction. In the case of dry reforming, the CO_2_ produced is the net balance, as CO_2_ is an input to the process.

**Table 3:** Advantages and disadvantages of hydrogen production technologies (modified from [27] and [5]).

Technology	Advantages	Disadvantages
Natural Gas Pyrolysis	No emission CO_2_ and CO Oxygen and water not required Fuel flexibility	Carbon formed High operating temperatures
Reforming of Methane	Most extensive industrial experience Oxygen not required Lowest process temperature Best H_2_/CO ratio for H_2_ production	Highest air emissions
Partial Oxidation of Methane	Reduced desulfurization requirement No catalyst requirement Low methane slip	Low H_2_/CO ratio High operating temperatures Complex handling process
Coal Gasification	Remove impurities before burning the fuel Lower material costs	High coal demands High CO_2_ production/H_2_
Electrolysis	No emission CO_2_ Production of high purity hydrogen	High energy requirement
Autothermal Reforming of Methane	Lower process temperature than partial oxidation Low methane slip	Limited commercial experience Air/oxygen requirement

**Table 4:** Low heat value (LHV) of pure substances ^2^ [32].

Substance	LHV (MJ/kg)
CH_4_	51.12
CO_2_	-
H_2_	120.00
CO	10.10
H_2_O	-
C (s)	32.80
O_2_	-

^2^ Pressure 1 atm, Temperature 20 °C.

**Table 5:** Standard mole chemical exergy of pure substances [34].

Substance	$Ex_{ch}\;(\mathrm{kJ}/\mathrm{mol})$
CH_4_	831.65
CO_2_	19.87
H_2_	236.10
CO	275.10
H_2_O	9.50
C (s)	410.00
O_2_	3.87

**Table 6:** Rate constants of fuel consumption in thermochemical processes under reference conditions.

	*Temperature (K)*	*Pressure (bar)*	$k\;\left(s^{-1}\right)$	$F_{CH_{4_{0}}}\left(kmol/h\right)\;$
Natural Gas Pyrolysis	1173	1	−1.58·10^−3^	1.23·10^−4^
Dry Reforming of Methane	973	1	−1.68·10^4^	6.18·10^−5^
Steam Reforming of Methane	1000	1	−0.10	7.90
Partial Oxidation of Methane	1223	1	−2.10·10^−3^	12.94
Coal Gasification	1123	1	−2.77·10^−4^	187.82
Autothermal Reforming of Methane	1510	21	−53.21	100.00

**Table 7:** Energy efficiencies for hydrogen production reactors.

	Energy Efficiency (%)			
	in Transformation DWSIM	in Transformation MATLAB	Reference [5,13,14,46]	Process DWSIM
Natural Gas Pyrolysis	58.99	55.19	~55–58	44.95
Dry Reforming of Methane	62.13	62.32	~56–85	40.85
Steam Reforming of Methane	71.98	71.66	~74	64.95
Partial Oxidation of Methane	81.27	81.47	~70–80	78.39
Coal Gasification	62.49	62.13	~60	51.75
Electrolysis	69.46	-	~50–70	47.64
Autothermal Reforming of Methane	60.66	61.38	−60–75	56.85

**Table 8:** Energy requirement and CO_2_ production for hydrogen production reactors.

	Energy Requirement (kWh/kg H_2_)	$\frac{mol\;CO_{2}}{mol\;H_{2}}$	$\frac{mol\;C}{mol\;H_{2}}$	$\frac{mol\;CO_{2}}{mol\;C}$
Natural Gas Pyrolysis	16.28	-	0.5	-
Dry Reforming of Methane	24.50	0.34	0.54	0.47
Steam Reforming of Methane	10.84	0.32	0.32	1.00
Partial Oxidation of Methane ^2^	-	4.48	4.48	1.00
Coal Gasification	3.76	0.77	0.77	1.00
Electrolysis	47.99	-	-	-
Autothermal Reforming of Methane	5.76	0.31	0.31	1.00

^2^ CO_2_ production as a function of H_2_ and energy produced.

**Table 9:** Exergy efficiencies simulated for hydrogen production reactors.

	$\eta_{exergy}\;\left(\%\right)$	$Ex_{Destruction}\left(\%\right)$	$Ex_{Exhaust}\left(\%\right)$	$Ex_{un-used}\left(\%\right)$
Natural Gas Pyrolysis	46.88	53.12	40.70	93.82
Dry Reforming of Methane	47.97	52.03	1.46	53.50
Steam Reforming of Methane	78.87	21.13	0.26	21.39
Partial Oxidation of Methane	58.35	41.65	5.51	47.16
Coal Gasification	50.92	49.08	6.45	55.55
Electrolysis	87.92	12.08	0.73	12.81
Autothermal Reforming of Methane	89.08	10.92	0.75	11.66

**Table 10:** Exergy efficiencies with and without CCS technology for hydrogen production reactors.

	Energy Efficiency (%)	
	in Transformation	With CCS
Natural Gas Pyrolysis	58.99	58.99
Dry Reforming of Methane	62.13	46.60
Steam Reforming of Methane	71.98	53.99
Partial Oxidation of Methane	81.27	60.95
Coal Gasification	62.49	46.87
Electrolysis	69.46	69.46
Autothermal Reforming of Methane	60.66	45.50

## Supplementary Materials

The following are available online at https://www.mdpi.com/1099-4300/22/11/1286/s1, Figure S1: Simulation for Natural Gas Pyrolysis in DWSIM, Figure S2: Simulation for Dry Reforming of Methane in DWSIM, Figure S3: Simulation for Steam Reforming of Methane in DWSIM, Figure S4: Simulation for Partial Oxidation of Methane in DWSIM, Figure S5: Simulation for Water Electrolysis in DWSIM, Figure S6: Simulation for Coal Gasification in DWSIM, Figure S7: Simulation for Auto-thermal Reforming of Methane in DWSIM, Table S1: Thermodynamic and rate constants.

## Nomenclature

$\eta_{Energy}$	Energy efficiency, %
$m_{i}$	Mass flow, kg s^−1^
LHV	Lower heating value, MJ kg^−1^
$Q_{Heat}$	Heat of reaction, MJ s^−1^
$\eta_{Exergy}$	Exergy efficiency, %
$Ex_{i}$	Exergy, kJ mol^−1^
P	Pressure, bar
T	Temperature, K
k	Kinetic constant, s^−1^
$F_{CH_{4_{0}}}$	Initial molar flow fuel, kmol h^−1^

## Abbreviations

GHG	Greenhouse Gas
IPCC	Intergovernmental Panel on Climate Change
NGP	Natural Gas Pyrolysis
DRM	Dry Reforming of Methane
SMR	Steam Reforming of Methane
POM	Partial Oxidation of Methane
CG	Coal Gasification
ATM	Auto-thermal Reforming of Methane
WE	Water Electrolysis
WGS	Water-Gas Shift
PrOx	Preferential Oxidation
RWGS	Reverse Water-Gas Shift
LHV	Lower Heating Value
CCS	Carbon Capture and Sequestration
