# Supplementary material for: Comparative Analysis of Energy and Exergy Performance of Hydrogen Production Methods
Source: Entropy (Basel). 2020 Nov 12;22(11):1286. doi: 10.3390/e22111286 (PMC7712718; doi:10.3390/e22111286)
Supplement: Supplementary file 1 [file entropy-22-01286-s001.pdf]

entropy

MDPI

Natural Gas Pyrolysis (NGP)

- *Chemical Reactions*

$$CH_4 \leftrightarrow C + 2H_2\,, \qquad \Delta H^0_{298} = +74{,}87\ kJ/mol$$

- *Kinetic*

$$r = \frac{kK_{CH_4}\left(P_{CH_4} - \frac{P^2_{H_2}}{K_P}\right)}{\left(1 + K_{CH_4}P_{CH_4} + \frac{1}{K_{H_2}}P^{1.5}_{H_2}\right)^2} \tag{S1}$$

Dry Reforming of Methane (DMR)

- *Chemical Reactions*

1) $CH_4 + CO_2 \leftrightarrow 2CO + 2H_2,\ \Delta H^0_{298} = +247kJ/mol$
2) $CO + H_2O \leftrightarrow H_2 + CO_2,\ \Delta H^0_{298} = +41.7kJ/mol$
3) $CH_4 \leftrightarrow C + 2H_2\,,\ \Delta H^0_{298} = +74{,}87\ kJ/mol$

- *Kinetic*

$$r_1 = \frac{k_1P_{CH_4}P_{CO_2}}{\left(K_{CO_2,1}P_{CO_2} + K_{CH_4,1}P_{CH_4}\right)}\left(1 - \frac{\left(P_{CO}P_{H_2}\right)^2}{K_{P_1}\left(P_{CH_4}P_{CO_2}\right)}\right) \tag{S2}$$

$$r_2 = \frac{k_2K_{CO_2,2}K_{H_2,2}P_{CO_2}P_{H_2}}{\left(1 + K_{CO_2,2}P_{CO_2} + K_{H_2,2}P_{H_2}\right)^2}\left(1 - \frac{\left(P_{CO}P_{H_2O}\right)}{K_{P_2}\left(P_{CO_2}P_{H_2}\right)}\right) \tag{S3}$$

$$r_3 = \frac{k_3K_{CH_4,3}\left(P_{CH_4} - \frac{P^2_{H_2}}{K_{P_3}}\right)}{\left(1 + K_{CH_4,3}P_{CH_4} + \frac{1}{K_{H_2,3}}P^{1.5}_{H_2}\right)^2} \tag{S4}$$

Steam Reforming of Methane (SMR)

- *Chemical Reactions*

1) $CH_4 + H_2O \leftrightarrow 3H_2 + CO,\ \Delta H^0_{298} = +206\ kJ/mol$
2) $CO + H_2O \leftrightarrow H_2 + CO_2,\ \Delta H^0_{298} = +41.7kJ/mol$
3) $CH_4 + 2H_2O \leftrightarrow 4H_2 + CO_2\,,\ \Delta H^0_{298} = +253.1\ kJ/mol$

- *Kinetic*

$$r_1 = \frac{\frac{k_1}{P^{2.5}_{H_2}}\left(P_{CH_4}P_{H_2O} - \frac{P^3_{H_2}P_{CO}}{K_1}\right)}{DEN^2} \tag{S5}$$

$$r_2 = \frac{\frac{k_2}{P_{H_2}}\left(P_{CO}P_{H_2O} - \frac{P_{H_2}P_{CO_2}}{K_2}\right)}{DEN^2} \tag{S6}$$

$$r_3 = \frac{\frac{k_3}{P^{3.5}_{H_2}}\left(P_{CH_4}P^2_{H_2O} - \frac{P^4_{H_2}P_{CO_2}}{K_3}\right)}{DEN^2} \tag{S7}$$

$$DEN = 1 + K_{CH_4}P_{CH_4} + K_{CO}P_{CO} + K_{H_2}P_{H_2} + \frac{K_{H_2O}P_{H_2O}}{P_{H_2}} \quad \text{(S8)}$$

Partial Oxidation of Methane (POM)

- *Chemical Reactions*
    1) $CH_4 + 2O_2 \rightarrow CO_2 + 2H_2O,\ \Delta H^0_{298} = -802\ kJ/mol$
    2) $CH_4 + H_2O \leftrightarrow 3H_2 + CO,\ \Delta H^0_{298} = +206\ kJ/mol$
    3) $CH_4 + CO_2 \leftrightarrow 2CO + 2H_2,\ \Delta H^0_{298} = +247 kJ/mol$

- *Kinetic*

$$r_1 = \frac{(k_1 P_{CH_4} P_{O_2}^2)}{\left(1 + K_{CH_4}P_{CH_4} + K_{O_2}\right)^2} \quad \text{(S9)}$$

$$r_2 = k_2 P_{CH_4} P_{H_2O}\left(1 - \frac{P_{H_2}P_{CO}^3}{K_2 P_{CH_4} P_{H_2O}}\right) \quad \text{(S10)}$$

$$r_3 = k_3 P_{CH_4} P_{CO_2}\left(1 - \frac{P_{H_2}^2 P_{CO}^2}{K_3 P_{CH_4} P_{CO_2}}\right) \quad \text{(S11)}$$

Water Electrolysis (WE)

- *Chemical Reactions*

$$H_2O \rightarrow 1/2O_2 + 2H_2$$

Coal Gasification (CG)

- *Chemical Reactions*
    1) $C + CO_2 \leftrightarrow 2CO,\ \Delta H^0_{298} = -542.1\ kJ/mol$
    2) $\mathrm{C} + \mathrm{H_2O} \leftrightarrow \mathrm{CO} + \mathrm{H_2},\ \Delta \mathrm{H}^0_{298}$ = -583.3 kJ/mol
    3) $CO + H_2O \leftrightarrow H_2 + CO_2,\ \Delta H^0_{298} = +41.7 kJ/mol$
- *Kinetic*

$$r_1 = k_1 P_{CO_2}^{0.31} \quad \text{(S12)}$$

$$r_2 = k_2 P_{H_2O}^{0.73} \quad \text{(S13)}$$

$$r_3 = \frac{\frac{k_3}{P_{H_2}}\left(P_{CO}P_{H_2O} - \frac{P_{H_2}P_{CO_2}}{K_3}\right)}{\left(1 + \frac{K_{H_2O}P_{H_2O}}{P_{H_2}} + K_{H_2}P_{H_2} + K_{CO}P_{CO}\right)^2} \quad \text{(S14)}$$

Autothermal Reforming of Methane (ATR)

- *Chemical Reactions*
    1) $CH_4 + H_2O \leftrightarrow 3H_2 + CO,\ \Delta H^0_{298} = +206\ kJ/mol$
    2) $CO + H_2O \leftrightarrow H_2 + CO_2,\ \Delta H^0_{298} = +41.7 kJ/mol$
    3) $CH_4 + 2H_2O \leftrightarrow 4H_2 + CO_2\ ,\ \Delta H^0_{298} = +253.1\ kJ/mol$
    4) $CH_4 + 2O_2 \rightarrow CO_2 + 2H_2O,\ \Delta H^0_{298} = -802\ kJ/mol$
- *Kinetic*

$$r_1 = \frac{\frac{k_1}{P_{H_2}^{2.5}}\left(P_{CH_4}P_{H_2O} - \frac{P_{H_2}^3 P_{CO}}{K_1}\right)}{DEN^2} \tag{S15}$$

$$r_2 = \frac{\frac{k_2}{P_{H_2}}\left(P_{CO}P_{H_2O} - \frac{P_{H_2} P_{CO_2}}{K_2}\right)}{DEN^2} \tag{S16}$$

$$r_3 = \frac{\frac{k_3}{P_{H_2}^{3.5}}\left(P_{CH_4}P_{H_2O}^2 - \frac{P_{H_2}^4 P_{CO_2}}{K_3}\right)}{DEN^2} \tag{S17}$$

$$r_4 = \frac{(k_4 P_{CH_4} P_{O_2}^2)}{\left(1 + K_{CH_4,4}P_{CH_4} + K_{O_2}\right)^2} \tag{S18}$$

$$DEN = 1 + K_{CH_4}P_{CH_4} + K_{CO}P_{CO} + K_{H_2}P_{H_2} + \frac{K_{H_2O}P_{H_2O}}{P_{H_2}} \tag{S19}$$

Table S1. Thermodynamic and rate constants. Constants are in [mol, kg, s, bar].

**Natural Gas Pyrolysis** [1]			
**Parameter**	**Value**	**Parameter**	**Value**
k	$6.95 \cdot 10^3 \exp\left(-\frac{58893}{RT}\right)$	K_{CH_4}	$0.21 \exp\left(-\frac{567}{RT}\right)$
K_{H_2}	$5.18 \cdot 10^7 \exp\left(-\frac{133210}{RT}\right)$	K_P	$2.98 \cdot 10^5 \exp\left(-\frac{84400}{RT}\right)$
**Dry Reforming of Methane** [1]			
**Parameter**	**Value**	**Parameter**	**Value**
k_1	$1.35 \cdot 10^{-8} \exp\left(-\frac{3115.22}{RT}\right)$	k_2	$0.35 \cdot 10^6 \exp\left(-\frac{81030}{RT}\right)$
k_3	$6.95 \cdot 10^3 \exp\left(-\frac{58893}{RT}\right)$	$K_{CO_2,1}$	$9.25 \cdot 10^{-8} \exp\left(+\frac{4883.32}{RT}\right)$
$K_{CH_4,1}$	$2.46 \cdot 10^{-7} \exp\left(+\frac{4606.68}{T}\right)$	$K_{CO_2,2}$	$0.5771 \exp\left(+\frac{9262}{RT}\right)$
$K_{H_2,2}$	$1.494 \exp\left(+\frac{6025}{RT}\right)$	$K_{CH_4,3}$	$0.21 \exp\left(-\frac{567}{RT}\right)$
$K_{H_2,3}$	$5.18 \cdot 10^7 \exp\left(-\frac{133210}{RT}\right)$	K_{P_1}	$6.78 \cdot 10^{14} \exp\left(-\frac{259660}{RT}\right)$
K_{P_2}	$56.4971 \exp\left(-\frac{36580}{RT}\right)$	K_{P_3}	$2.98 \cdot 10^5 \exp\left(-\frac{84400}{RT}\right)$
**Steam Reforming of Methane** [2]			
**Parameter**	**Value**	**Parameter**	**Value**
k_1	$4.22 \cdot 10^{15} \exp\left(-\frac{240100}{RT}\right)$	k_2	$1.96 \cdot 10^6 \exp\left(-\frac{67130}{RT}\right)$
k_3	$1.02 \cdot 10^{15} \exp\left(-\frac{243900}{RT}\right)$	K_{CH_4}	$6.65 \cdot 10^{-4} \exp\left(+\frac{38280}{RT}\right)$
K_{H_2O}	$1.77 \cdot 10^5 \exp\left(-\frac{88680}{RT}\right)$	K_{H_2}	$6.12 \cdot 10^{-9} \exp\left(+\frac{82900}{RT}\right)$

Parameter	Value	Parameter	Value
K_{CO}	$8.23 \cdot 10^{-5} \exp\left(+\frac{70650}{RT}\right)$	K_1	$\exp\left(30.42 - \frac{27106}{T}\right)$
K_2	$\exp\left(-3.798 + \frac{4160}{T}\right)$	K_3	$\exp\left(34.218 - \frac{31266}{T}\right)$
**Partial Oxidation of Methane** [3,4]			
**Parameter**	**Value**	**Parameter**	**Value**
k_1	$1.10 \exp\left(+\frac{166000}{RT}\right)$	k_2	$4.19 \cdot 10^{-9} \exp\left(+\frac{29000}{RT}\right)$
k_3	$2.42 \cdot 10^{-9} \exp\left(+\frac{23700}{RT}\right)$	K_{CH_4}	$6.65 \cdot 10^{-4} \exp\left(+\frac{103500}{RT}\right)$
K_{O_2}	$1.77 \cdot 10^{-5} \exp\left(+\frac{66200}{RT}\right)$	K_2	$5.75 \cdot 10^{12} \exp\left(-\frac{95411}{RT}\right)$
K_3	$7.24 \cdot 10^{10} \exp\left(-\frac{179960}{RT}\right)$		
**Coal Gasification** [5,6]			
**Parameter**	**Value**	**Parameter**	**Value**
k_1	$2.07 \cdot 10^{7} \exp\left(-\frac{220000}{RT}\right)$	k_2	$1.12 \cdot 10^{8} \exp\left(-\frac{245000}{RT}\right)$
k_3	$1.96 \cdot 10^{6} \exp\left(-\frac{67130}{RT}\right)$	K_{H_2O}	$1.77 \cdot 10^{5} \exp\left(-\frac{88680}{RT}\right)$
K_{H_2}	$6.12 \cdot 10^{-9} \exp\left(+\frac{82900}{RT}\right)$	K_{CO}	$8.23 \cdot 10^{-5} \exp\left(+\frac{70650}{RT}\right)$
K_3	$\exp\left(-3.798 + \frac{4160}{T}\right)$		
**Auto-thermal Reforming of Methane** [7]			
**Parameter**	**Value**	**Parameter**	**Value**
k_1	$1.955 \cdot 10^{6} \exp\left(-\frac{67130}{RT}\right)$	k_2	$1.02 \cdot 10^{15} \exp\left(-\frac{243900}{RT}\right)$
k_3	$5.852 \cdot 10^{17} \exp\left(-\frac{204000}{RT}\right)$	k_4	$1.10 \exp\left(+\frac{166000}{RT}\right)$
K_{CH_4}	$6.65 \cdot 10^{-4} \exp\left(+\frac{38280}{RT}\right)$	K_{CO}	$8.23 \cdot 10^{-5} \exp\left(+\frac{70650}{RT}\right)$
K_{H_2}	$6.12 \cdot 10^{-9} \exp\left(+\frac{82900}{RT}\right)$	K_{H_2O}	$1.77 \cdot 10^{5} \exp\left(-\frac{88680}{RT}\right)$
$K_{CH_4,4}$	$6.65 \cdot 10^{-4} \exp\left(+\frac{103500}{RT}\right)$	K_{O_2}	$1.77 \cdot 10^{-5} \exp\left(+\frac{66200}{RT}\right)$
K_1	$5.75 \cdot 10^{12} \exp\left(-\frac{11476}{RT}\right)$	K_2	$1.26 \cdot 10^{-2} \exp\left(+\frac{4639}{RT}\right)$
K_3	$7.242 \cdot 10^{10} \exp\left(-\frac{21646}{RT}\right)$		

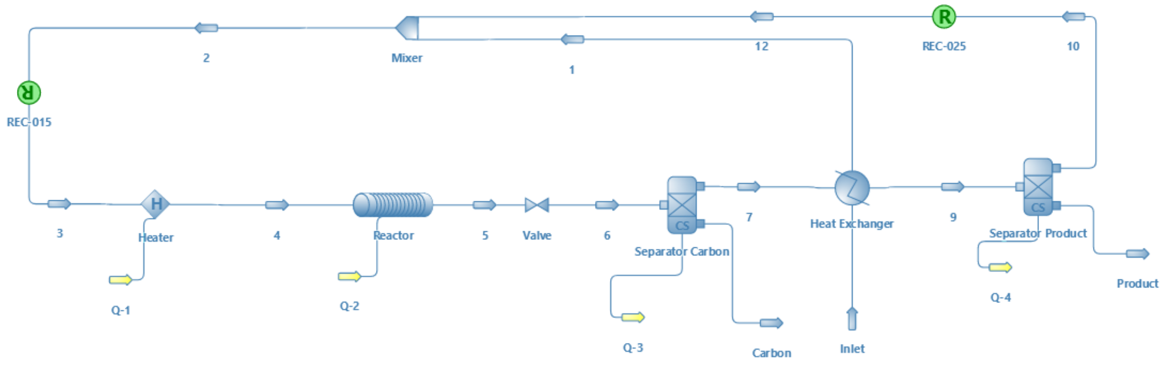

Figure S1. Simulation for Natural Gas Pyrolysis in DWSIM.

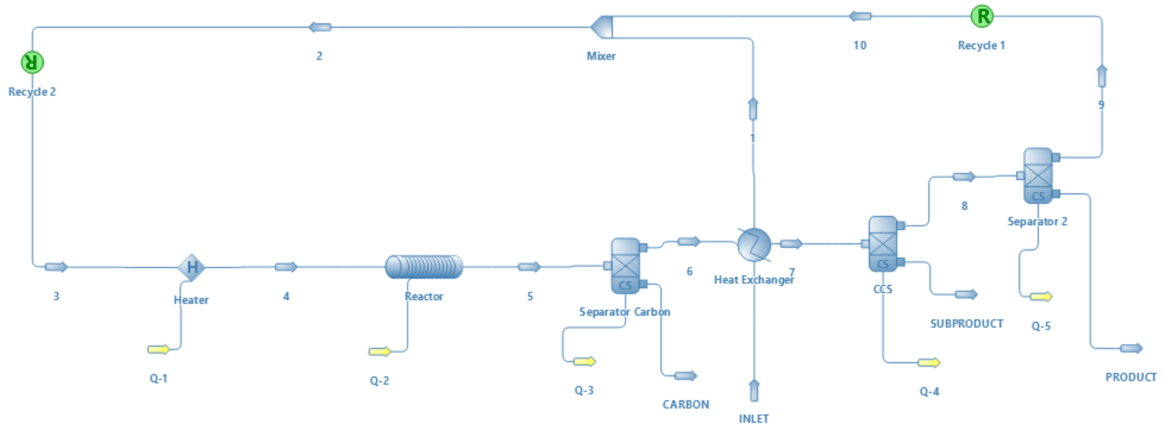

Figure S2. Simulation for Dry Reforming of Methane in DWSIM.

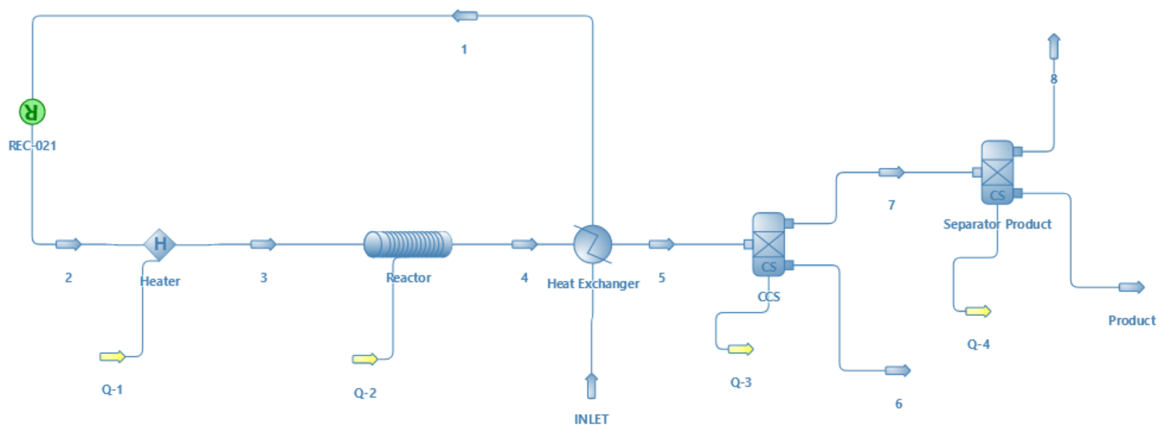

Figure S3. Simulation for Steam Reforming of Methane in DWSIM.

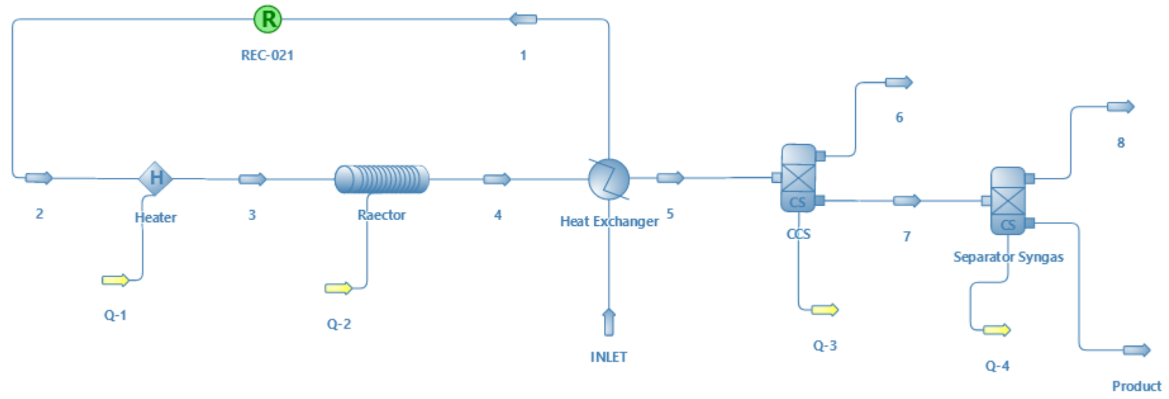

Figure S4. Simulation for Partial Oxidation of Methane in DWSIM.

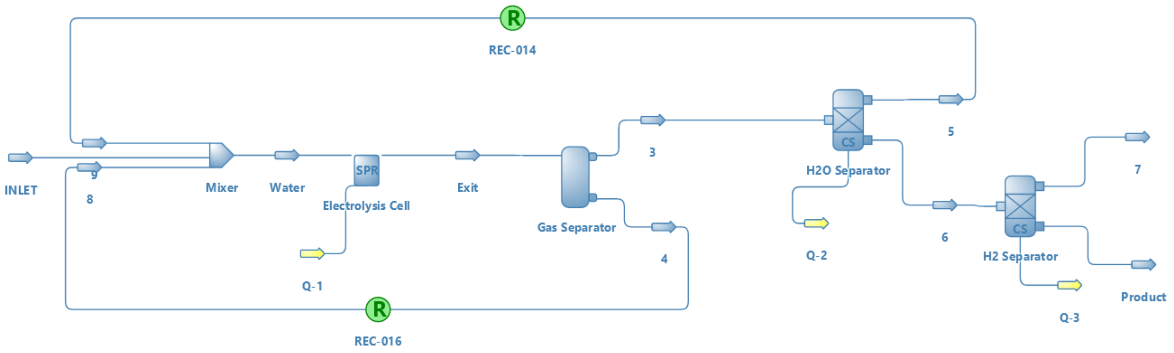

Figure S5. Simulation for Water Electrolysis in DWSIM.

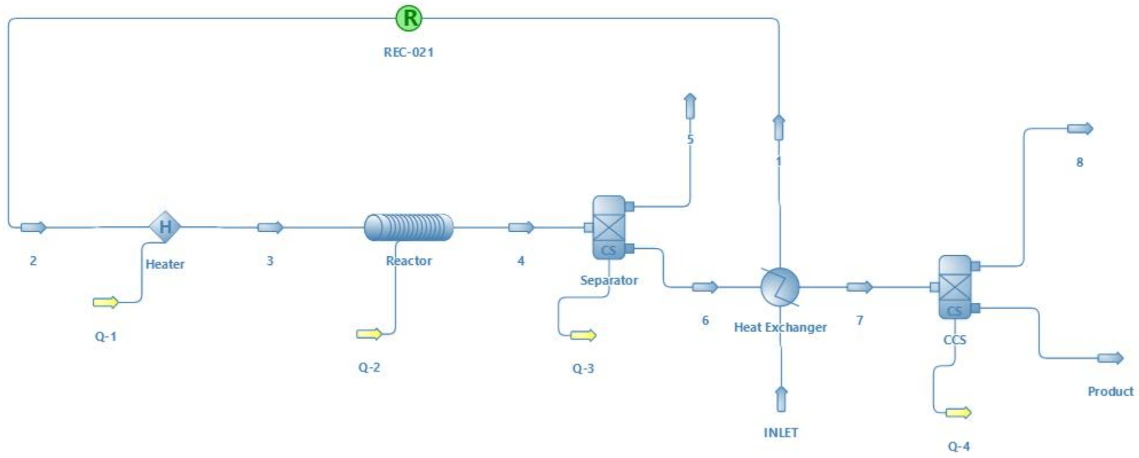

Figure S6. Simulation for Coal Gasification in DWSIM.

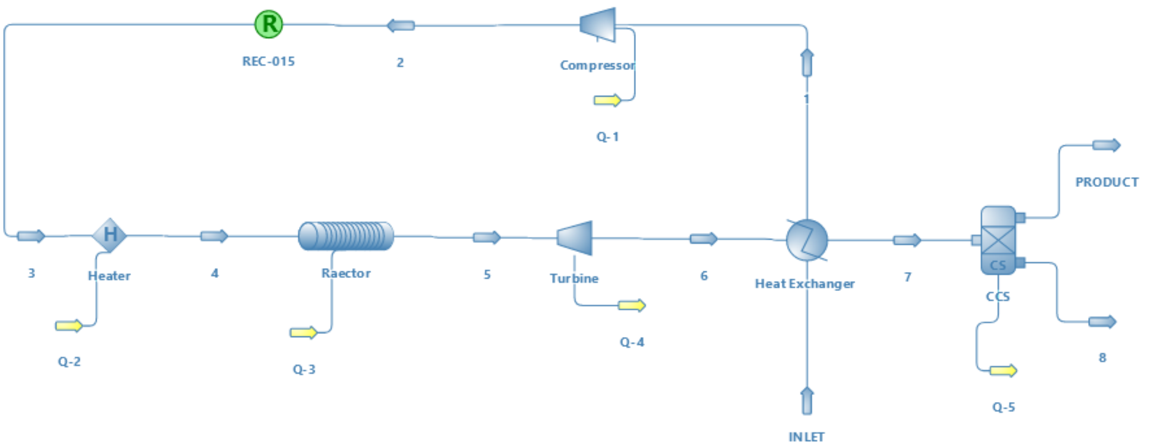

Figure S7. Simulation for Auto-thermal Reforming of Methane in DWSIM.

1. Benguerba, Y.; Virginie, M.; Dumas, C.; Ernst, B. Methane dry reforming over Ni-Co/Al2O3: Kinetic modelling in a catalytic fixed-bed reactor. *Int. J. Chem. React. Eng.* **2017**, *15*.
2. Keith, J.M. Hydrogen Education Curriculum Path at Michigan Technological University. **2010**.
3. Hoang, D.L.; Chan, S.H.; Ding, O.L. Kinetic modelling of partial oxidation of methane in an oxygen permeable membrane reactor. *Chem. Eng. Res. Des.* **2005**, *83*, 177–186.
4. Fernandes, F.A.N.; Sousa, J.F.; Souza, C.P.; Rodrigues, S. *Modeling of partial oxidation of methane in a membrane reactor*; 2005;
5. Umeki, K.; Yamamoto, K.; Namioka, T.; Yoshikawa, K. High temperature steam-only gasification of woody biomass. *Appl. Energy* **2010**, *87*, 791–798.
6. Solli, K.-A.; Kumar Thapa, R.; Moldestad, B.M.E. Screening of Kinetic Rate Equations for Gasification Simulation Models. *Proc. 9th EUROSIM Congr. Model. Simulation, EUROSIM 2016, 57th SIMS Conf. Simul. Model. SIMS 2016* **2018**, *142*, 105–112.
7. Zahedi nezhad, M.; Rowshanzamir, S.; Eikani, M.H. Autothermal reforming of methane to synthesis gas: Modeling and simulation. *Int. J. Hydrogen Energy* **2009**, *34*, 1292–1300.

© 2020 by the authors. Submitted for possible open access publication under the terms and conditions of the Creative Commons Attribution (CC BY) license (http://creativecommons.org/licenses/by/4.0/).
